# Integrating multi-type features and knowledge graph for graded prediction of drug-induced liver injury in humans

**DOI:** 10.1371/journal.pcbi.1013640

**Published:** 2026-07-14

**Authors:** Ying Liu, Kaimiao Hu, Jie Geng, Qi Dai, Leyi Wei, Ran Su

**Affiliations:** 1 School of Computer Software, College of Intelligence and Computing, Tianjin University, Tianjin, China; 2 Department of Cardiology, Tianjin Chest Hospital, Tianjin University, Tianjin, China; 3 Tianjin Key Laboratory of Cardiovascular Emergency and Critical Care, Tianjin Municipal Science and Technology Bureau, Tianjin, China; 4 College of Life Science and Medicine, Zhejiang Sci-Tech University, Hangzhou, China; 5 Center for Artificial Intelligence driven Drug Discovery, Faculty of Applied Science, Macao Polytechnic University, Macao SAR, China; University of Zurich, Division of Infectious Diseases and Hospital Epidemiology, Raemistrasse 100, SWITZERLAND, Zurich, ZH, 8091

## Abstract

Drug-induced liver toxicity poses a threat to human health and remains a significant reason for drug withdrawal from the market. Therefore, early identification of drug-induced liver injury (DILI) during drug development is crucial. However, most studies on hepatotoxicity prediction are limited to single type of features or binary toxicity assessment. In this study, we propose a novel liver toxicity prediction model called MolFPKG-DILI (Molecular Graph, FingerPrint and Knowledge Graph-based DILI), which integrates multi-type compound features and knowledge graph for assessing DILI severity. Molecular fingerprints and molecular graphs capture different information of compounds, and models using individual features alone have shown limited performance. Our model incorporates an attention mechanism to effectively fuse the information from molecular fingerprints and molecular graphs. Furthermore, we leverage the relationship between drugs and other entities from the knowledge graph to achieve liver toxicity grading. Experimental results demonstrate that our proposed method exhibits highly competitive performance in both DILI/No-DILI and Most-DILI/Less-DILI classification. External validation conducted on an independent set of benchmark drugs yields satisfactory results, demonstrating the robustness of our approach. Additionally, we employ a series of interpretability methods to investigate the relationship between the different types of data utilized by the model and toxicity outcomes. These analyses highlight the interpretability of our method, providing valuable insights and support for drug toxicity evaluation.

## Author summary

Drug-induced liver injury (DILI) is a critical concern in drug development, often resulting in the withdrawal of numerous medications due to their hepatotoxic effects. Traditional methods of assessing DILI are not only costly but also labor-intensive. While recent computational strategies leveraging molecular fingerprints and descriptors have enhanced efficiency, they typically rely on single data types. This reliance can neglect the complex interactions among various molecular features, thereby constraining the predictive accuracy and effectiveness of these models. Our research introduces MolFPKG-DILI, an advanced model that integrates multiple feature sources—molecular fingerprints, molecular graphs, and expansive knowledge graphs with diverse drug-related data—to enhance prediction accuracy and detail. This model not only predicts the presence of DILI but also discerns varying degrees of liver injury severity. Utilizing attention-based feature fusion and relational graph convolutional networks, MolFPKG-DILI outperforms existing models, offering nuanced insights into DILI levels. External validation on 17 drugs and a series of interpretable analyses have demonstrated the competitiveness of the model in practical applications. This method is anticipated to serve as a dependable tool for toxicity risk assessment in early drug development, reducing the likelihood of development failures attributed to liver toxicity.

## Introduction

Drug-induced liver injury (DILI) is a prevalent adverse reaction in clinical practice, ranging from mild elevation of liver enzymes to severe liver dysfunction and even liver failure, posing a significant threat to life [1]. Studies have shown that between 1990 and 2010, a total of 133 drugs were removed from the market due to safety concerns, approximately 27.1% of which were due to liver toxicity [2]. Therefore, it is crucial to assess the potential hepatotoxicity risk of drugs during the clinical development and application of drugs. Researchers commonly employ in vivo and in vitro experiments as well as animal studies to assess liver toxicity. However, these methods are time-consuming, costly [3], and the occurrence rate of idiosyncratic DILI in clinical studies is only around 0.1% or even lower [4]. Currently, the field of drug development is facing a dilemma of high investment with low returns, and there is an urgent need for new approaches to address the issue of predicting drug-induced hepatotoxicity.

In recent years, an increasing number of studies have started to use computer technology to investigate drug-induced liver injury, providing relatively reliable predictions of DILI while reducing both financial and time costs. The most commonly used method in this field is based on predicting DILI using molecular fingerprints or chemical descriptors of drugs as features. Molecular fingerprints use fixed-length vectors to represent molecular structures, where each bit position indicates the presence of a particular substructure or functional group. Molecular descriptors (MDs) refer to the numerical representation of the physical and chemical information of drug molecules, covering details such as molecular weight, charge, and lipophilicity [5]. Ai et al. [6] used drug simplified molecular input line entry system (SMILES) to get molecular fingerprint profiles and constructed 36 classifiers using three machine learning algorithms and 12 types of molecular fingerprints, ultimately obtaining the best ensemble model composed of five base classifiers. Li et al. [7] used Mold2, Mol2vec, and the Molecular ACCess System (MACCS) fingerprint as drug descriptor features, and combined machine learning techniques with neural networks to propose DeepDILI (deep learning-powered DILI), further improving the accuracy of liver toxicity prediction. However, these features may not capture more complex structural characteristics within drug molecules, and these methods are unable to handle drug molecules with new structural components.

In addition, some studies have also used drug molecular graph for DILI prediction. Compounds are inherently graph-structured, composed of atoms as nodes and chemical bonds as edges. These graph-based representations can potentially capture more detailed structural characteristics compared to fingerprint-based features. For instance, Wu et al. [8] extracted molecular representations from a pretrained three-dimentional spatial structure-based graph neural networks (GNN) model, and built an interpretable liver toxicity prediction model called GeoDILI. Lim et al. [4] used supervised subgraph mining methods to extract molecular graph features, further improving the prediction accuracy of DILI. The graph-based approaches can better capture the complex structural information within drug molecules, and are also able to handle drug molecules with new structural components. Additionally, some studies [9,10] have employed equivariant graph neural networks (E-GNN) to extract 3D structural features from various molecules, successfully achieving tasks such as de novo drug design and predicting protein-ligand interactions, yielding promising results. This approach provides a valuable reference for leveraging the 3D structural characteristics of drugs.

Molecular fingerprints pay more attention to the overall physicochemical properties of the drug, whereas molecular graphs encompass information regarding the connections between different atoms. By using both features, a more comprehensive representation of information can be obtained, thus better addressing the issue of liver toxicity prediction. Gu et al. [11] integrated molecular descriptor features into molecular graph representations and utilized a series of graph learning methods to predict DILI; Cai et al. [12] proposed the FP-GNN model, which combines molecular graphs and molecular fingerprints to predict drug properties such as toxicity and lipophilicity. But the above-mentioned methods simply concatenate the two types of features without considering that different features may contribute differently to liver toxicity prediction. He et al. [13] addressed the drug-drug interaction (DDI) problem by using molecular fingerprints as node features and drug pair association information as edge features, which were then input into a relational graph convolutional network (RGCN). They employed multi-head attention mechanisms to facilitate multi-level predictions of drug combinations and associated risks. This approach combines structural characteristics with interaction network information, offering new insights for feature design in liver toxicity prediction tasks.

Recently, some studies have employed knowledge graph (KG) data to address issues such as predicting adverse drug reaction (ADR) and DDIs. By incorporating entities such as indications, targets, genes, etc., knowledge graphs enrich the representation of drug nodes from different perspectives. Bean et al. [14] constructed a knowledge graph consisting of four types of nodes (drugs, protein targets, indications, and adverse reactions), utilizing machine learning algorithms to achieve the first-ever prediction of unknown ADRs. In other fileds, Chen et al. [15] proposed MSKG-DDI, which combines drug molecular graphs and knowledge graph information, utilizing multimodal features for DDI prediction. He et al. [16] defined multiple edge types, including drug-disease associations and disease similarity, and utilized a multi-aggregate graph convolutional network combined with a graph transformer to process heterogeneous graphs. This approach achieved a high performance in drug-disease association prediction. The pathogenesis and clinical phenotypes of DILI are complex and diverse, involving multiple organs and functions of the body. Leveraging knowledge graph to identify DILI allows for effective utilization of information related to other drug entities. This approach not only holds promise for achieving better DILI prediction results but also facilitates a deeper understanding of the mechanisms and manifestations of DILI, providing robust support for clinical treatment.

In spite of the positive results achieved by the aforementioned studies in predicting DILI, there are still some limitations and shortcomings. For instance:

- Existing methods mostly rely on single type of features to predict DILI. Models using multiple types of features often resort to simple concatenation without fully exploring the relationships between them to derive the most suitable features for DILI prediction.
- Currently, there is a lack of research focusing on using knowledge graph to predict hepatotoxicity. The methods for feature extraction from KG data are relatively straightforward and do not capitalize on the characteristics of knowledge graph to choose more appropriate models.
- Moreover, the majority of studies have only achieved binary classification of whether a drug has hepatotoxicity or not, without further distinguishing the severity of toxicity levels.

The toxicity of a drug is not only determined by its molecular structural features but is also significantly influenced by dosage and pharmacokinetic (PK) parameters. For instance, acetaminophen is deemed safe at therapeutic doses; however, in cases of overdose, it can lead to liver failure due to the accumulation of the toxic metabolite N-acetyl-p-benzoquinone imine (NAPQI), which arises from metabolic saturation [17]. Pharmacokinetic parameters such as maximum plasma concentration (*C*_*max*_) and clearance (CL) directly affect the exposure level and distribution patterns of the drug within the body, thereby determining the likelihood of toxicity occurrence [18]. Although dose determines toxicity in clinical practice, the primary goal of the model proposed in this study is to make a preliminary prediction of the intrinsic hepatotoxicity of drugs based on molecular information. In future studies, drug dose-response data and pharmacokinetic parameters will be integrated to further improve the predictive power and practical applicability of the model.

**Fig 1:**
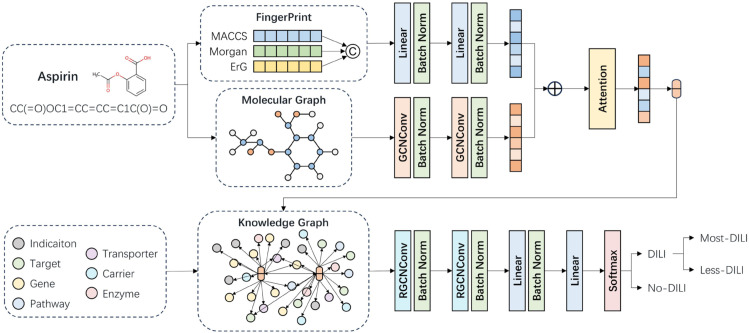
Overall flowchart of the method. First, MACCS, the Morgan fingerprint (also known as Extended Connectivity Fingerprints, ECFP), and the Pharmacophore ErG fingerprint, as well as the corresponding molecular graphs, were generated according to the compounds. The concatenated molecular fingerprints were input into a fully connected model to obtain processed fingerprint features. Concurrently, the molecular graphs were input into a graph convolutional network (GCN) model to derive more representative graph features. The attention mechanism was employed to fuse these two types of features, dynamically adjusting their respective contributions. Subsequently, knowledge graph was gathered, and the fused features were utilized as drug node attributes. By leveraging the RGCN model, knowledge graph information was extracted, enabling the classification and grading of hepatotoxicity.

In this study, we introduce a novel deep learning architecture, MolFPKG-DILI (Molecular Graph, FingerPrint and Knowledge Graph-based DILI), which integrates multi-type features of drugs to achieve graded prediction of drug-induced liver injury. The overall view of the proposed method is shown in Fig 1. The architecture is primarily divided into data collection, molecular fingerprint feature extraction, molecular graph feature extraction, attention-based feature fusion, knowledge graph feature extraction, and hepatotoxicity prediction. The model first learns features from various molecular fingerprints and molecular graphs, and employs an attention mechanism to effectively fuse these two types of features. Subsequently, we collected drug-related entities from multiple databases to construct a knowledge graph consisting of 7 entity types, 5414 entities, and 34,676 relationships. Using the fused features as the drug node features in the knowledge graph, we leverage RGCN to learn the relational features, ultimately enabling the prediction of DILI severity levels. The experimental results demonstrate that our model outperforms several previous models in predicting toxicity and severity and holds promise as an important tool for drug development and assessment of DILI severity. In addition, an external validation on 17 drugs was conducted, and the results suggest the strong generalization capability of our model. Furthermore, MolFPKG-DILI exhibits interpretability across three types of data, which could assist researchers in performing more comprehensive clinical drug risk assessments. The implementation of the proposed method is available at https://github.com/RanSuLab/MolFPKG-DILI.

## Materials and methods

### Data collection

We collected drugs related to DILI from the DILIrank [19] and DILIst [20] databases. In DILIrank, compounds are categorized based on their DILI risk into four classes: Most-DILI-concern (192 drugs), Less-DILI-concern (278 drugs), No-DILI-concern (312 drugs), and Ambiguous DILI-concern (254 drugs). DILIst classifies compounds into two categories: DILI positives (768 drugs) and DILI negatives (511 drugs). We initially selected DILI-negative drugs from the DILIst, along with Most-DILI-concern and Less-DILI-concern drugs from the DILIrank. Subsequently, drugs without retrievable SMILES were excluded, resulting in 477 No-DILI and 453 DILI (including 182 Most-DILI and 271 Less-DILI). Association edges between drugs and other entities were extracted from four databases to construct a drug-related knowledge graph (see Sect 2.5.1 of Materials and methods for details). To ensure each drug had associated edges, relationship data from these four databases were matched against the collected drugs, with those lacking any association edges excluded. Ultimately, 824 drugs were obtained, comprising 404 No-DILI and 420 DILI (267 Less-DILI and 153 Most-DILI). Notably, in predicting strong and weak toxicity, to ensure data balance, we randomly selected an equal number of Less-DILI drugs to match the Most-DILI drugs for training, resulting in 153 Most-DILI and 153 Less-DILI drugs. Finally, we summarize the division of the training and test sets, along with the number of compounds in each category, in Table 1.

**Table 1:** The data distribution of the training set and test set under the two classification tasks.

	No-DILI	DILI	Less-DILI	Most-DILI	Total
All data	404	420	153	267	824
All data-train	323	336	/	/	659
All data-test	81	84	/	/	165
DILI data	/	/	153	153	306
DILI data-train	/	/	122	122	244
DILI data-test	/	/	31	31	62

### Molecular fingerprint feature extraction

Molecular fingerprints cover the local structural information of molecules, providing a simple and effective representation of their physicochemical properties. Each type of feature within these fingerprints is mutually independent, making them an excellent tool for characterizing molecular properties. In this study, we selected three types of molecular fingerprints: MACCS [21], Morgan [22], and the Pharmacophore ErG fingerprint [23]. These methods describe molecular features from different dimensions, ensuring a representative and comprehensive depiction of the molecules.

The MACCS fingerprint contains functional groups and substructure motifs of compounds, comprising 166 bits. When writing Python code, the first bit is retained as a placeholder, resulting in an actual 167-dimensional vector. The Morgan fingerprint is a type of circular fingerprint, and the ErG fingerprint encompasses chemical or functional interaction features observed in drug-receptor interactions, reflecting the physicochemical properties of compounds. We denote the MACCS fingerprint as $F\;P_{maccs}(x)$, the Morgan fingerprint as $F\;P_{morgan}(x)$, and the Pharmacophore ErG fingerprint as $F\;P_{erg}(x)$. By concatenating these three fingerprint features, we obtain the input to the molecular fingerprint model FPNN (fingerprint neural network): $F\;P(x)=[F\;P_{maccs}(x),F\;P_{morgan}(x),F\;P_{erg}(x)]$ , where *x* represents the compound. It is known that the forward pass of a fully connected layer is given by y=f(wa+b), where *a* is the input, *y* is the output, *w* represents the weight matrix, *b* is the bias vector, and *f* is the activation function. Similarly, for the FPNN, we can derive:

$$\text{FPNN}(x)=f(W_{2}*f(W_{1}*F\;P(x)+b_{1})+b_{2})$$ (1)

where FPNN(x) is the model output, *W*_1_, *W*_2_, *b*_1_ and *b*_2_ represent the weight matrices and bias vectors of two fully connected layers, respectively, and * denotes matrix multiplication. The activation function *f* is *ReLU*, which operates as f(x)=max(0,x). This module extracts features from the high-dimensional molecular fingerprint vectors, capturing the physicochemical information of the drugs, thus facilitating subsequent integration with molecular graph features.

### Molecular graph feature extraction

Inspired by the methods employed by Cai et al. [12] in their work for processing molecular graphs, we extracted eight features for each atom in the compound. These features included atomic number, degree, charge, chirality tag, number of surrounding hydrogen atoms, hybridization, aromatic ring, and atomic mass. These features were one-hot encoded and used as atomic node features, resulting in a dimensionality of 133. Based on the distribution of chemical bonds between atoms, an adjacency matrix was generated to construct the molecular graph. The molecular graph not only encompasses the overall structural information of the molecule but also considers unique atomic information such as charge and mass.

We represent the molecular graph as G=(V,A), where *V* represents each atom in the molecular graph, and *A* represents the adjacency matrix generated above. We use MGCN (molecular graph convolutional network) to extract representative features of molecular graphs, which contains two layer of GCN. The core of MGCN involves transforming node features between two convolutional layers, for which the adjacency matrix needs to be symmetrically normalized to balance the differences in node degrees and ensure the stability of feature values. The updated feature of each node includes information from both the node itself and its neighboring nodes. We show this as:

$$x_{i}^{\left(l+1\right)}=\sigma \left(\sum_{j\in V_{i}}{\hat{D}}^{-\frac{1}{2}}\hat{A}{\hat{D}}^{-\frac{1}{2}}x_{j}^{\left(l\right)}W^{\left(l\right)}+b^{\left(l\right)}\right)$$ (2)

where $x_{i}^{\left(l+1\right)}$ represents the feature of node *i* at layer l+1, $x_{j}^{\left(l\right)}$ represents the feature of node *j* at layer *l*, σ denotes a non-linear activation function, $\hat{A}$ is the adjacency matrix *A* with added self-loops, $\hat{D}$ denotes the degree matrix corresponding to $\hat{A}$, $V_{i}$ represents all neighboring nodes of node *i*, including *i* itself, *W^(l)^* is the weight matrix at layer *l*, and *b^(l)^* is the bias at layer *l*. If we use *X* to represent the node features of the molecular graph, the feature transformation process of MGCN can be simplified as:

$$\text{MGCN}(X)=\mathrm{softmax}(\hat{A}\mathrm{ReLU}(\hat{A}XW^{\left(0\right)}+b^{\left(0\right)})W^{\left(1\right)}+b^{\left(1\right)})$$ (3)

### Attentional feature fusion

From the above two modules, we obtained the processed molecular fingerprint features FPNN(x) and molecular graph features MGCN(x). These two features represented different dimensional information of drug molecules. Considering their varying contributions to the subsequent classification task, we used an attention mechanism for feature fusion. The goal of this module was to obtain the fusion ratio of the two features, which can be simply expressed as:

$$\text{ATT}(x)=\alpha_{\text{FPNN}}\cdot \text{FPNN}(x)+\alpha_{\text{MGCN}}\cdot \text{MGCN}(x)$$ (4)

where $\alpha_{\text{FPNN}}$ and $\alpha_{\text{MGCN}}$ represented the attention coefficients of the two features, respectively. Specifically, for compound *x*_*i*_, with molecular fingerprint features $\text{FPNN}(x_{i})$ and molecular graph features $\text{MGCN}(x_{i})$, a feedforward neural network with a *tanh* activation function was used to obtain:

$$w_{\text{FPNN}}=q^{T}\cdot \tanh(w\cdot (\text{FPNN}(x_{i}){)}^{T}+b)$$ (5)

$$w_{\text{MGCN}}=q^{T}\cdot \tanh(w\cdot (\text{MGCN}(x_{i}){)}^{T}+b)$$ (6)

where *w* was the weight matrix, *b* was the bias vector, and *q* was the shared attention vector. The attention values were regularized using *softmax* to obtain the attention coefficients, as shown in the following equations:

$$\alpha_{\text{FPNN}}=\mathrm{softmax}(w_{\text{FPNN}})=\frac{\exp(w_{\text{FPNN}})}{\exp(w_{\text{FPNN}})+\exp(w_{\text{MGCN}})}$$ (7)

$$\alpha_{\text{MGCN}}=\mathrm{softmax}(w_{\text{MGCN}})=\frac{\exp(w_{\text{MGCN}})}{\exp(w_{\text{FPNN}})+\exp(w_{\text{MGCN}})}$$ (8)

The final feature representation was obtained by weighted fusion of the two features using the attention coefficients.

### Knowledge graph feature extraction and hepatotoxicity prediction

#### Knowledge graph construction.

The knowledge graph consists of 8 types of nodes and 8 types of relationships (Table 2). The nodes include drug, indication, pathway, gene, target, enzyme, carrier, and transporter. The edges comprise drug_drug, has_indication, has_pathway, has_gene, has_target, has_enzyme, has_carrier, and has_transporter relationships.

**Table 2:** The edge and the source of the edge contained in the knowledge graph.

Network	Data Source	Meaning
drug_drug	Calculated by Tanimoto score	The similarity between the two drugs.
has_indication	SIDER database [24]	The information on marketed medicines and their recorded adverse drug reactions.
has_target	DrugBank database [25]	The relationship between drugs and targets, that is, the drug exhibits binding affinity or interacts with specific molecular or cellular targets within the organism.
has_pathway	Drug-Path database [26]	The drug-induced pathways predicted from drug-induced gene expression data based on the Connectivity Map.
has_gene	DGIdb database [27]	Drug-gene interactions, which meaning the potential interactions or effects that drugs may have on specific genes or gene products.
has_enzyme	DrugBank database [25]	The relationship between drugs and enzymes. Enzyme is a protein which catalyzes chemical reactions involving a given drug.
has_transporter	DrugBank database [25]	The relationship between drugs and transporters. Transporter is a membrane bound protein which shuttles ions, small molecules or macromolecules across membranes.
has_carrier	DrugBank database [25]	The relationship between drugs and carriers. Carrier is a secreted protein which binds to drugs, carrying them to cell transporters.

The drug_drug relationship is determined based on the Tanimoto score. Using the previously obtained Morgan molecular fingerprints, we calculated the Tanimoto scores between pairs of drugs. The score threshold is treated as a hyperparameter, and comparative experiments are conducted to evaluate its impact in S2 Text. The remaining relationships are primarily collected from four drug-related databases: the SIDER database [24], which contains adverse drug reactions and indications for 1,284 drugs; the Drug-Path database [26], which includes relationships between 1,824 drugs and 93 pathways; the DGIdb database [27], which provides 14,218 drug-gene relationships; and the DrugBank database [25], which contains multiple entities such as targets, enzymes, carriers, and transporters, offering various drug-related relationships.

By matching the collected drug data with the edges from the aforementioned databases, we ultimately identified 34,676 edges related to 824 drugs. The details of entities and associations for all drugs and drugs with DILI are shown in Tables 3 and 4.

**Table 3:** The details of entities and associations in the knowledge graph for all drugs.

	Indication	Target	Pathway	Gene	Enzyme	Transporter	Carrier	Total
Drug	630	711	364	745	546	401	225	824
Entity	2113	1016	93	1918	215	130	39	5414
Relation	9055	4118	3739	13728	2409	1276	351	34676

**Table 4:** The details of entities and associations in the knowledge graph for drugs with DILI.

	Indication	Target	Pathway	Gene	Enzyme	Transporter	Carrier	Total
Drug	237	267	135	286	226	180	110	306
Entity	1421	559	89	1433	150	74	16	3684
Relation	4380	1503	1386	6483	1186	622	171	15731

#### Knowledge graph module and toxicity prediction.

We utilized RGCN networks to learn information from knowledge graphs that aids in toxicity classification. Subsequently, we used two fully connected layers to achieve hepatotoxicity classification. The input module is a knowledge graph *G*, where the drug node features are denoted as ATT(x), and the features of other nodes are randomly generated. The output is a label if a drug is toxic or non-toxic.

RGCN is an extension of GCN that can handle heterogeneous graph information. Unlike GCN, RGCN assigns different weight matrices to each type of relation, considering the influence of different types of relations on the nodes. Specifically, the RGCN layer first computes the output using node features and the weight matrix for the specified relation type, then aggregates all relation types to obtain the updated node representation. The node feature update in GCN can be simplified as:

$$x_{i}^{\left(l+1\right)}=\sigma \left(\sum_{j\in V_{i}}\frac{1}{c_{i}}x_{j}^{\left(l\right)}W^{\left(l\right)}\right)$$ (9)

where *c*_*i*_ is a normalization factor, and the bias vector is omitted. For RGCN, let *R* represent the set of relations in the knowledge graph. The node feature update is given by:

$$x_{i}^{\left(l+1\right)}=\sigma \left(x_{i}^{\left(l\right)}W_{0}^{\left(l\right)}+\sum_{r\in R}\sum_{j\in V_{i}^{r}}\frac{1}{c_{i,r}}x_{j}^{\left(l\right)}W^{\left(r\right)}\right)$$ (10)

where $V_{i}^{r}$ denotes the set of neighbor nodes of node *i* under relation r∈R, and *c*_*i*,*r*_ is the corresponding normalization factor. Subsequently, we use two fully connected neural network layers to classify the drug nodes, with the final layer employing a *sigmoid* activation function to output the binary classification result. The overall training process is evaluated using the binary cross-entropy loss function.

To address the research objective of grading drug hepatotoxicity, we have specifically divided it into two subtasks: No-DILI vs DILI (Task 1) and Less-DILI vs Most-DILI (Task 2), with independent models trained for each to meet their respective classification goals. Specifically, Task 1 aims to determine if a drug is hepatotoxic, using all drug data as input; Task 2 focuses on classifying the severity of hepatotoxicity among these toxic drugs, with input limited to the drugs labeled DILI in the original data. The two models are independent of each other and can both be trained independently to achieve the prediction target.

### Experiment setup and evaluation metrics

The molecular fingerprints used in this study were obtained using the Rdkit package. Specifically, MACCS fingerprints were generated via the GetMACCSKeysFingerprint method in the Rdkit.AllChem module; Morgan fingerprints were obtained using the GetMorganFingerprintAsBitVect method with a radius of 2 and 1024 bits; and ErG fingerprints were generated using the GetErGFingerprint method.

Model performance was evaluated using five-fold cross-validation, with stratified random sampling in each fold to ensure that the proportion of each category matched that of the original dataset, thereby effectively addressing data imbalance. All models were constructed based on the PyTorch framework and trained using the Adam optimizer. To effectively mitigate overfitting, a dropout rate of 0.5 was applied to the FPNN, MGCN, RGCN, and final fully connected layers. Additionally, we utilized the early stopping strategy to obtain the final prediction results.

Both models share an identical architecture, comprising the fingerprint module, molecular graph module, attention fusion module, and knowledge graph module. However, two models differ slightly in training hyperparameters. The learning rate for the non-toxicity model is 0.001, while for the strong/weak toxicity model it is 0.0001. The regularization coefficient is set to 0.01, and batch size to 16 for both models. All hyperparameters (including number of layers, hidden size, learning rate, batch size, etc.) were optimized using grid search on the validation set to achieve the best performance.The random seed was set to 5 during the dataset splitting stage, and set to 1 for model initialization and other operations involving randomness.

All model training was conducted on a server equipped with four NVIDIA GTX 1080Ti GPUs. To ensure reproducibility and consistency of results, the following key software packages and libraries were used for data processing, model training, and result visualization: matplotlib (v3.9.4), numpy (v1.26.4), pandas (v2.2.3), rdkit (v2024.3.3), scikit-learn (v1.6.1), pytorch (v2.6.0), torch_geometric (v2.4.0), and visdom (v0.2.4). All experiments were strictly conducted using the specified versions above.

To comprehensively evaluate the model’s performance, we utilized various metrics such as Accuracy, Precision, Sensitivity, Specificity, F1-score, and Area Under Curve (AUC). The calculation formulas for these metrics are as follows:

$$\text{Accuracy}=\frac{\text{TP}+\text{TN}}{\text{TP}+\text{TN}+\text{FP}+\text{FN}}$$ (11)

$$\text{Precision}=\frac{\text{TP}}{\text{TP}+\text{FP}}$$ (12)

$$\text{Sensitivity}=\frac{\text{TP}}{\text{TP}+\text{FN}}$$ (13)

$$\text{Specificity}=\frac{\text{TN}}{\text{TN}+\text{FP}}$$ (14)

$$\text{F1-score}=\frac{2\cdot \text{TP}}{2\cdot \text{TP}+\text{FP}+\text{FN}}$$ (15)

$$\text{AUC}=\int_{0}^{1}\text{TPR}({\text{FPR}}^{-1}(t))dt$$ (16)

where $\text{TPR}=\frac{\text{TP}}{\text{TP}+\text{FN}}$, and $\text{FPR}=\frac{\text{FP}}{\text{TN}+\text{FP}}$. TP, TN, FP and FN represent true positive, true negative, false positive and false negative respectively, *t* is the threshold.

## Results and discussion

### Chemical space analysis

**Fig 2:**
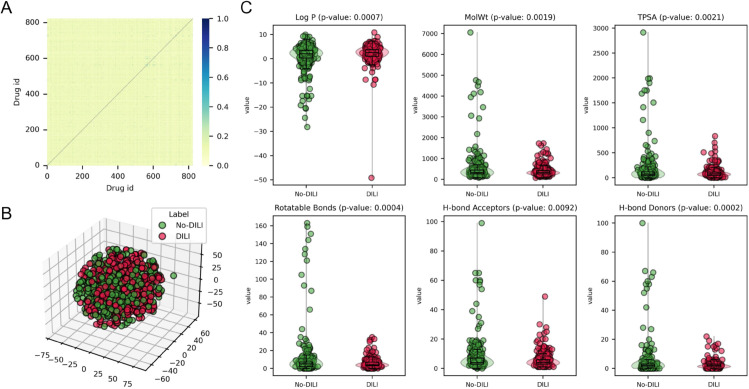
Three different perspectives on the diversity of the compounds. (A) Tanimoto similarity heatmap of all compounds using Morgan fingerprint. (B) The t-SNE distribution of the compounds labeled DILI and No-DILI. (C) Physicochemical property distributions of compounds with DILI and No-DILI.

To gain a comprehensive understanding of the compounds involved in this study, we conducted data analysis and visualization of both toxic and non-toxic drugs. The Tanimoto coefficient is commonly used to measure the similarity between compounds, and we utilized this coefficient to generate a similarity heatmap of the compounds. As shown in Fig 2A, it primarily appears yellow, indicating low similarity among the compounds in the dataset and highlighting the chemical structural diversity. We employed the t-SNE (t-Distributed Stochastic Neighbor Embedding) algorithm to map the high-dimensional drug Morgan fingerprints into a three-dimensional space for drug visualization. From Fig 2B, it can be observed that toxic and non-toxic drugs are intermingled without clear boundaries, indicating that toxicological classification is not a straightforward task. Lastly, we conducted statistical analysis on six physicochemical properties of the compounds, namely LogP (the Wildman Crippen log P value), MolWt (the average molecular weight of the molecule), TPSA (topological polar surface area), rotatable bonds (the number of rotatable bonds), H-bond acceptors (the number of hydrogen bond acceptors), and H-bond donors (the number of hydrogen bond donors). In statistics, a p-value< 0.001 indicates significant differences, while p> 0.05 implies insignificant differences. From Fig 2C, it can be observed that there are substantial differences between toxic and non-toxic drugs in terms of LogP, Rotatable Bonds, and H-Bond Donors. Toxic drugs generally exhibit stronger lipophilicity, as well as a higher number of rotatable bonds and hydrogen bond donors. The absence of properties with p> 0.05 suggests a certain level of compound diversity. For a detailed analysis of the properties of strong and weak toxic drugs, please refer to the S1 Fig.

### Ablation study

**Fig 3:**
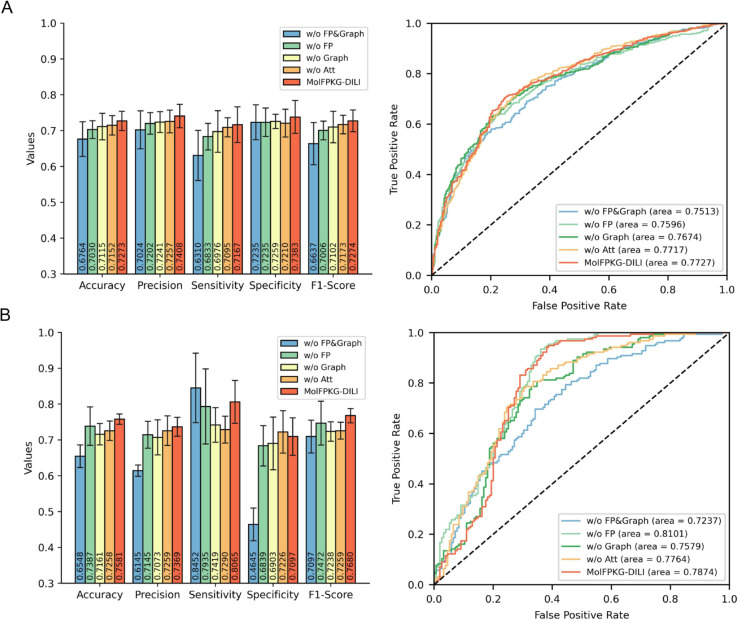
Ablation results on the variants of MolFPKG-DILI. (A) The accuracy and ROC curves (receiver operating characteristic curves) of the MolFPKG-DILI variants on DILI and No-DILI data. (B) The accuracy and ROC curves of the MolFPKG-DILI variants on Most-DILI and Less-DILI data.

To validate the effectiveness of the various modules added in this model and the features used, we conducted experiments with several model variants as shown in Table 5. The variants of MolFPKG-DILI as follows: (1) removing the molecular fingerprint and molecular graph encoding module (w/o FP&Graph), (2) removing the molecular fingerprint encoding module (w/o FP), (3) removing the molecular graph encoding module (w/o Graph), (4) removing the attention feature fusion module (w/o Att), and (5) Using all of the components as a baseline for comparison of ablation experiments (MolFPKG-DILI). As previously mentioned, our model is developed based on the drug knowledge graph to perform the hepatotoxicity grading prediction task. Both molecular fingerprints and molecular graphs serve as the features of drug nodes within the knowledge graph. Therefore, in the design of ablation experiments, all models are implemented based on the knowledge graph. For the “w/o FP&Graph” model, the features are randomly generated vectors of specified dimensions. The experimental results using these model variants are shown in Fig 3. In terms of the binary classification of toxicity, the MolFPKG-DILI model outperformed other model variants in all evaluation metrics. Regarding the classification of strong and weak toxicity, although some model variants achieved better results in certain metrics, the majority of indicators still demonstrated the superior performance of the MolFPKG-DILI model.

**Table 5:** Variants of model in ablation study.

	Attention	Fingerprint	Molecular graph
w/o FP&Graph	×	×	×
w/o FP	×	×	✓
w/o Graph	×	✓	×
w/o Att	×	✓	✓
MolFPKG-DILI	✓	✓	✓

#### Analysis of ablation studies on attention feature fusion.

The objective of this section is to demonstrate the efficacy of the added attention feature fusion module in the model for toxicity classification. The MolFPKG-DILI model and the w/o model were trained on the same data splits and utilized the same cross-validation methodology. As shown in Fig 3A, the MolFPKG-DILI model achieved an ACC of 0.7273 in the binary toxicity classification, while the w/o Att model obtained an ACC of 0.7152. Furthermore, all evaluation metrics of the MolFPKG-DILI model were higher than those of the w/o Att model. For Most-DILI and Less-DILI data, except for specificity where w/o Att slightly outperformed MolFPKG-DILI, the remaining indicators supported the effectiveness of the attention module. The ROC curves of the two models are depicted in Fig 3B, with MolFPKG-DILI exhibiting a slightly higher AUC than w/o Att and positioned in the top-left area, indicating improved classification performance after incorporating the attention module.

#### Analysis of ablation studies on molecular feature types.

In order to validate the optimal performance achieved by utilizing both fingerprint and molecular graph features in the model, we conducted ablation experiments by excluding each type of feature individually and both types of features simultaneously. As shown in Fig 3, the w/o FP & Graph model performed the worst in both binary toxicity prediction and strong/weak toxicity prediction. However, the performance improved to varying degrees with the addition of any one of the compound features, indicating that random features of the w/o FP & Graph model have limited utility in toxicity classification and compound-specific information is necessary. Fig 3A indicates that the model using both features outperformed the single-feature models, suggesting complementarity between molecular fingerprint features and molecular graph features in binary toxicity classification. In the case of strong/weak toxicity classification, the w/o FP model exhibited highly competitive performance. This can be observed from the ROC curves in Fig 3B, where the predictions of w/o FP even surpassed w/o Att. This indicates that molecular graph features are effective in capturing subtle differences between strong and weak toxic compounds. However, MolFPKG-DILI still outperformed w/o FP in most metrics. Overall, the use of multiple types of features proves beneficial for toxicity classification, and combining them with the attention feature fusion module achieves optimal performance.

### Comparative experiment with baselines

The baseline models employed in this study can be categorized into four groups: (1) models utilizing only molecular fingerprint features, which include SVM(Support Vector Machine), RF(Random Forest), FPNN, DeepDILI [7], and 2020-DILI-CNN-MFE [28]; (2) models utilizing only molecular graph features, which include GCN (Graph Convolutional Network), GAT (Graph Attention Network) [29], GraphSAGE (Graph Sample and Aggregation) [30] and DILIGeNN [31]; (3) models combining both molecular fingerprint and molecular graph features, represented by FP-Graph; and (4) models using only knowledge graph, which include KGDNN [32], KG-SVM and KG-RF. Below is a brief introduction to each method, and please refer to S1 Text for more detailed information. The experimental scripts of baselines are available at https://github.com/RanSuLab/MolFPKG-DILI.

- SVM, RF: SVM is a supervised learning algorithm that finds an optimal hyperplane in the dataset to classify the data into different categories. RF is an ensemble learning algorithm that constructs multiple decision trees and aggregates their predictions through voting or averaging.
- FPNN: FPNN is essentially a multilayer perceptron (MLP) that learns from the given feature vectors through nonlinear transformations performed by multiple fully connected layers to obtain prediction results. In this study, four fully connected layers were utilized, with the model taking three types of molecular fingerprint concatenated features as input.
- DeepDILI [7]: DeepDILI incorporates the idea of ensemble learning by training five traditional machine learning algorithms (KNN, LR, SVM, RF, XGBoost) using a neural network framework, and obtains model-level representations of these five algorithms.
- 2020-DILI-CNN-MFE [28]: This method combines the concept of word embedding from natural language processing (NLP) and utilizes convolutional neural networks (CNN) and molecular fingerprint-embedded features to achieve DILI classification.
- GCN, GAT [29], GraphSAGE [30]: GCN performs convolutions on graph data to extract features and make predictions for nodes. GAT, unlike GCN, incorporates attention mechanisms to model relationships between nodes in the graph, enabling better capturing of complex node relationships. GraphSAGE aggregates neighbor node information to update node representations, with different weights assigned to different nodes, making it suitable for processing large-scale graph data.
- DILIGeNN [31]: This method leverages molecular optimization to build a custom graph dataset enriched with chemical features (e.g., bond lengths, partial charges) and applies multiple GNN architectures (GCN, GAT, GraphSAGE and Graph Isomorphism Network [33]) for DILI and molecular property prediction tasks.
- FP-Graph: This method leverages both molecular fingerprint and molecular graph features. It employs FPNN and GCN to learn from the molecular fingerprint and molecular graph features, respectively, concatenates the processed features from the two modules, and feeds them into an MLP classifier consisting of four fully connected layers to obtain the final results.
- KGDNN [32]: This method relies on knowledge graph. It employs the Node2Vec algorithm to generate embedding features for nodes and designs a deep neural network to perform drug node classification.
- KG-SVM, KG-RF: This method processes knowledge graph in the same way as KGDNN, and the classification methods are changed to SVM and RF.

Detailed toxicological classification results for the baseline models are presented in Tables 6 and 7. All comparative experiments were conducted based on five-fold cross-validation using the same stratified random sampling method. All models utilized the same dataset. In the classification of DILI and No-DILI, each fold containing 659 drugs in the training set and 165 drugs in the test set. In the classification of Most-DILI and Less-DILI, each fold containing 244 drugs in the training set and 62 drugs in the test set.

**Table 6:** Overall performance of MolFPKG-DILI and baseline methods on DILI and No-DILI data.

Model	Accuracy	Precision	Sensitivity	Specificity	F1-Score
SVM	0.6715	0.6803	0.6762	0.6667	0.6765
RF	0.6691	0.6673	0.7048	0.6321	0.6840
FPNN	0.6303	0.6363	0.6405	0.6198	0.6369
DeepDILI	0.6796	0.6327	**0.8857**	**0.4653**	**0.7381**
2020-DILI-CNN-MFE	0.6485	0.6102	0.8571	0.4321	0.7129
GCN	0.6582	0.6540	0.7024	0.6123	0.6760
GAT	0.6594	0.6721	0.6524	0.6667	0.6578
GraphSAGE	0.6824	0.6891	0.6929	0.6716	0.6897
DILIGeNN	0.6522	0.7084	0.5542	**0.7564**	**0.6199**
FP-Graph	0.6424	0.6388	0.6905	0.5926	0.6622
KGDNN	0.6448	0.6677	0.6762	0.6123	0.6587
KG-SVM	0.6982	0.6806	0.7738	0.6198	0.7232
KG-RF	0.6921	0.7020	0.6905	0.6938	0.6956
MolFPKG-DILI	**0.7273**	**0.7408**	**0.7167**	**0.7383**	**0.7274**

**Table 7:** Overall performance of MolFPKG-DILI and baseline methods on Most-DILI and Less-DILI data.

Model	Accuracy	Precision	Sensitivity	Specificity	F1-Score
SVM	0.6774	0.6566	0.7677	0.5871	0.7054
RF	0.6645	0.6606	0.6968	0.6323	0.6732
FPNN	0.5774	0.5988	0.5419	0.6129	0.5177
DeepDILI	0.5728	0.5620	0.7333	0.4059	0.6363
2020-DILI-CNN-MFE	0.5645	0.5625	0.5806	0.5484	0.5714
GCN	0.5935	0.6088	0.5419	0.6452	0.5960
GAT	0.5968	0.5999	0.5742	0.6194	0.5816
GraphSAGE	0.5903	0.5950	0.5677	0.6129	0.5764
DILIGeNN	0.5867	0.6211	0.2467	**0.9267**	**0.3454**
FP-Graph	0.6129	0.6144	0.6645	0.5613	0.6316
KGDNN	0.6742	0.6897	0.6710	0.6774	0.6754
KG-SVM	0.7226	0.7132	0.7548	0.6903	0.7310
KG-RF	0.6806	0.6879	0.6645	0.6968	0.6722
MolFPKG-DILI	**0.7581**	**0.7369**	**0.8065**	**0.7097**	**0.7680**

#### Comparison of model classification performance for different data types.

To evaluate the effectiveness of the knowledge graph, this study compared the classification performance of three data representation models: molecular fingerprints, molecular graphs, and knowledge graphs. As demonstrated by the results of toxicity experiments (as shown in Table 6), the model utilizing the knowledge graph outperformed both the molecular fingerprints and molecular graph data overall in this task. The sensitivity of KG-SVM reached as high as 0.7738, with an F1-score of 0.7232, representing the best-performing combination among all methods. Furthermore, for vector-type data such as molecular fingerprints and processed knowledge graphs, both SVM and RF algorithms were found to perform better in all classification tasks. In the classification tasks for strong and weak toxicity, a similar observation was noted where models based on knowledge graphs exhibited superior performance compared to the other two data types (as illustrated in Table 7). The classification performance of KG-SVM significantly surpassed that of other methods; in contrast, KG-RF and KGDNN demonstrated slightly inferior performance but still outperformed certain models utilizing molecular fingerprints and molecular graph data. This result robustly validates the advantages of knowledge graph in toxicity classification tasks, as the complex relationships and potential semantics encompassed within this data are crucial for enhancing classification performance.

#### Comparison of classification performance between MolFPKG and other baselines.

From Tables 6 and 7, it can be observed that MolFPKG-DILI exhibits the best performance for both classification tasks. In the toxicity classification, MolFPKG-DILI achieves the highest accuracy of 0.7273, which is 4.17% higher than the best baseline model. Compared to models solely utilizing molecular fingerprint, molecular graph, or knowledge graph, significant improvements in accuracy are observed. Moreover, among the four models that utilize molecular graph, the performance of DILIGeNN is lower than that of the remaining models. This indicates that the molecular graph features we have selected are more effective for the toxicity classification task. Additionally, in the toxicity severity experiment, MolFPKG-DILI demonstrates excellent performance with an accuracy of 0.7581, surpassing other baselines by at least 4.91%. Based on these experiments, using a single type of feature alone did not yield satisfactory classification results, and models utilizing molecular fingerprint and molecular graph features also proved to be inadequate. However, as demonstrated in the previous ablation experiment, the combination of molecular graph features and knowledge graph exhibited highly competitive results. This suggests that the approach of integrating knowledge graph with other commonly used compound features has achieved significant progress in liver toxicity classification research.

### Visualization of the prediction results

**Fig 4:**
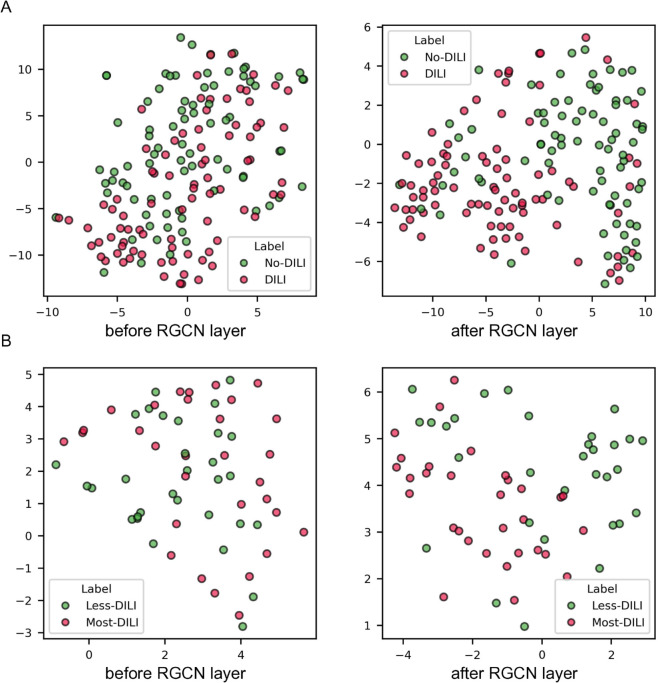
The t-SNE visualization of features. (A) shows the features before and after the RGCN layer, respectively, with results based on DILI and No-DILI data. (B) shows the features before and after the RGCN layer, respectively, with results based on Most-DILI and Less-DILI data.

In this section, we visualize the fused features and the features after the RGCN layer in a low-dimensional space using the t-SNE algorithm. Fig 4A shows the feature visualization results for the DILI and No-DILI, while Fig 4B shows the feature visualization results for Most-DILI and Less-DILI. It can be observed from the figures that the samples with fused features are mixed together without clear boundaries. However, after passing through the RGCN layer, the samples are well separated between class 0 and class 1. This provides a qualitative indication that the model may have learned separable feature representations for these sample groups. Combined with the classification accuracy and other evaluation metrics of the models in Table 6 and 7, this indicates that our model has good discriminative ability for samples with and without toxicity, as well as for samples with strong and weak toxicity.

### Independent validation

To further validate the effectiveness of MolFPKG-DILI in predicting drug-induced liver injury, we selected the remaining drugs from the DILIst dataset as an independent validation set. The specific construction process is as follows: First, by comparing the DILIst and DILIrank datasets, we identified toxic drugs that are uniquely present in the DILIst dataset. Subsequently, we utilized LiverTox [34] to search for definitive evidence of liver toxicity associated with these drugs, excluding drugs that may be classified as Ambiguous-DILI. We further categorized the toxicity labels of these drugs according to the liver toxicity grading criteria from the DILIrank dataset. Eventually, we obtained 13 DILI drugs, comprising 7 Less-DILI and 6 Most-DILI. For No-DILI drug samples, we integrated the latest data from relevant databases of the knowledge graph and got 4 non-toxic drugs from the DILIst dataset. For the data collection process of the validation set, please refer to the S4 Text.

Next, we evaluated the MolFPKG-DILI model using drugs in independent validation set, and the prediction results are listed in Table 8. We classified drugs as DILI if the prediction probability was over 0.5; otherwise, drugs were classified as No-DILI. The same rule applies in the classification of Most-DILI and Less-DILI. Regarding the classification of drug toxicity, MolFPKG-DILI correctly predicted all No-DILI drugs, and the accuracy reached 0.8235. In the case of strong/weak toxicity classification, all drugs except desflurane were predicted correctly. The above results indicated that our model can well identify the severity of drug-induced liver injury. In the independent validation set, although the MolFPKG-DILI model demonstrated high accuracy in predicting drug-induced hepatotoxicity, the predicted results of the model may need to be further confirmed in practical applications by incorporating dosage and pharmacokinetic data.

**Table 8:** The evaluation results of the MolFPKG-DILI model using drugs in independent validation set.

Drugs	Labels for DILI and No-DILI	Prediction	Prediction Probability	Labels for Most-DILI and Less-DILI	Prediction	Prediction Probability
colistimethate	0	0	0.9562	-	-	-
penicillin v	0	0	0.6963	-	-	-
polymyxin b sulfate	0	0	0.7463	-	-	-
gadobenate dimeglumine	0	0	0.7773	-	-	-
abiraterone	1	1	0.8787	1	1	0.5888
boceprevir	1	1	0.9932	1	1	0.6703
crizotinib	1	0	0.4608	1	1	0.5990
floxuridine	1	1	0.8676	1	1	0.7427
halothane	1	1	0.7427	1	1	0.6081
telaprevir	1	1	0.9005	1	1	0.6282
buprenorphine	1	1	0.7834	0	0	0.7541
desflurane	1	1	0.6199	0	1	0.3725
plicamycin	1	1	0.6606	0	0	0.9829
rivaroxaban	1	0	0.4706	0	0	0.5818
tibolone	1	1	0.5384	0	0	0.7599
trabectedin	1	0	0.2424	0	0	0.6215
flurbiprofen	1	1	0.5319	0	0	0.7191

It should be noted that the drug hepatotoxicity labels used in this study are derived from public datasets. These labels are based on expert consensus assessments of clinical case reports, which have comprehensively taken into account the actual dosage ranges and inter-individual exposure differences. Therefore, the toxicity risk predicted by the model essentially reflects the relative risk level of the drug at conventional therapeutic doses. However, since dose and pharmacokinetic information were not directly incorporated in this study, the model’s predictions may be biased in certain specific scenarios. In practical applications, it is necessary to further validate the model’s predictions in combination with information such as drug dosage.

To further validate the predictive stability of the model across different drug categories, we also classified the drugs based on ATC (Anatomical Therapeutic Chemical) codes and analyzed the performance differences of the model within each subgroup. Detailed results can be found in S3 Text.

### The interpretation of MolFPKG-DILI

Traditional deep learning models are often regarded as black boxes, making it challenging to comprehend their internal mechanisms. To address this issue, we conducted detailed interpretability case studies on three types of data employed by the model and their corresponding modules. By this means, we aim to reveal the underlying logic of the model’s decision-making process, thereby enhancing the transparency and reliability of the prediction results.

#### The interpretation based on fingerprint.

**Fig 5:**
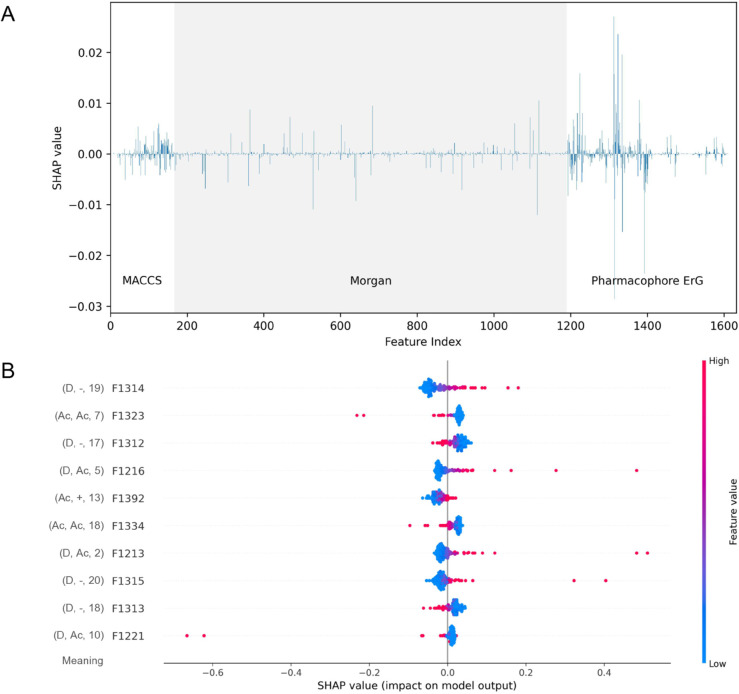
Interpretability analysis of fingerprint. (A) presents the SHAP values for all fingerprint data, and (B) provides a summary plot of the top 10 fingerprint features.

In this study, we introduced the SHapley Additive exPlanations (SHAP) method [35] to quantify the impact of molecular fingerprint features on predictive outcomes. The distribution of SHAP values for the three types of molecular fingerprint data used in the model is illustrated in Fig 5A, where positions 0-166 correspond to MACCS fingerprints, positions 167-1190 correspond to Morgan fingerprints, and positions 1191-1631 correspond to ErG fingerprints. We observed that all three fingerprint types contained multiple high-importance feature positions, indicating their significant role in the task of hepatotoxicity prediction. Notably, the ErG fingerprints exhibited the highest proportion of high-importance bits, contributing most substantially to the model’s predictions.

Fig 5B presents the distribution of the top 10 fingerprint features, with the X-axis representing the SHAP values. Positive and negative values indicate positive and negative contributions to the predictive outcome, respectively. For features F1314, F1216, F1392, F1213, and F1315, the presence of these features in a molecule resulted in positive SHAP values, suggesting a tendency to classify the drug as toxic. Conversely, features F1323, F1312, F1334, F1313, and F1221 exhibited the opposite conclusion, indicating that their presence may lead to classifying the drug as non-toxic. The five fingerprint positions significantly associated with hepatotoxicity identified in this study are all derived from ErG molecular fingerprints. The computational principle of ErG molecular fingerprints is based on the abstract processing of molecular structures: it extracts 6 types of core pharmacophore features, including hydrogen bond donors (D), hydrogen bond acceptors (Ac), hydrophobic endcaps (Hf), aromatic rings (Ar), positive charge centers (+), and negative charge centers (-), while ignoring specific atomic types and scaffold details. The shortest topological bond lengths between any two feature points are calculated using reduced graphs [23]. Therefore, each fingerprint position does not correspond to a specific molecular structure but rather represents the fuzzy quantification of "pharmacophore feature pair-topological distance".For example, F1314 corresponds to “(D, -, 19)”, indicating that hydrogen bond donors and negative charge centers are connected by 19 chemical bonds within the molecule, reflecting the topological synergy between these two features over long distances.

Wu et al. [8] previously constructed a DILI prediction model based on molecular geometric representation and identified seven substructures significantly associated with DILI. In this study, ErG fingerprint calculations were further performed on these substructures. The results showed that the substructure “NC1C(=O)N2C(=CCSC12)” exhibited significant values at fingerprint positions F1213 and F1392, while the substructure “CC1=C(C(=O)O)N2C(=O)CC2SC1” showed notable contributions at F1314, F1315, and F1392. These findings indicate a clear correspondence between the significant fingerprint positions identified by SHAP analysis and the toxicity-related substructures reported in previous studies, providing strong support for the reliability of the results in this study. Through the aforementioned SHAP analysis, we gained insights into the roles of various molecular fingerprint features in hepatotoxicity prediction, providing important reference points for subsequent drug development and safety assessment efforts.

#### The interpretation based on molcular graph.

To investigate the focal points of the model regarding molecular graph data, we utilized the Saliency method [36] to compute the importance of nodes and edges and performed visualizations. Specifically, we first calculated the gradients of the input molecular graph features with respect to the toxic class, resulting in an importance score for each node. And then, the importance scores of connected nodes were aggregated to derive the corresponding edge scores. This score can be considered as the model’s attention level towards the molecular graph data.

The study by Wu et al. [8] identified seven substructures of compounds significantly associated with DILI using various metrics. Based on this finding, we compared these substructures with our data. In this process, we discovered that two drugs were explicitly associated with the chlorobenzene moiety, and two drugs exhibited an association with the aniline moiety. Chlorobenzene is oxidized by CYP450 enzymes to generate epoxides, which can bind to proteins, DNA, and RNA, thereby inducing hepatocellular damage [8]. Additionally, clinical cases [37] have shown that acute high-dose exposure to chlorobenzene may trigger hepatotoxicity in humans. Aniline exerts toxic effects on primary cultured hepatocytes by inducing oxidative stress and apoptotic pathways, with mechanisms closely associated with the accumulation of reactive oxygen species (ROS), mitochondrial dysfunction, and DNA damage [38]. There have been multiple reports of liver damage caused by aniline [39].

**Fig 6:**
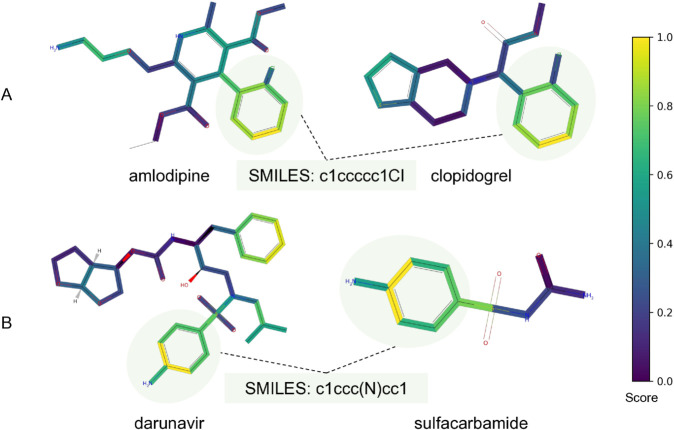
Interpretability analysis of molcular graph. The importance scores of the drugs related to the (A) substructure “c1ccccc1Cl” and (B) substructure “c1ccc(N)cc1”. The color of the edge depends on its importance score.

We visualized the molecular graph data of these four drugs. As shown in the Fig 6, the model assigned higher attention weights (marked in yellow-green) to these key substructures, while the remaining parts were represented in darker colors. This result not only demonstrates the interpretability of the model but also indicates that MolFPKG-DILI is capable of capturing important substructural information related to hepatotoxicity from complex drug structures.

#### The interpretation based on knowledge graph.

To evaluate whether MolFPKG-DILI can effectively leverage knowledge graph, we focused on the relationships of the top 100 drugs with the highest prediction accuracy to demonstrate their clinical relevance and interpretability. First, we obtained prediction accuracies for all drugs through five-fold cross-validation and selected the top 100 drugs. This result comprised 52 toxic and 48 non-toxic drugs, indicating the model’s capability to effectively distinguish between DILI and No-DILI compounds. Subsequently, we identified entities connected to these drugs in the knowledge graph, ranked them by frequency of occurrence in descending order, and selected the top 20 entities. The detailed information of the top 20 entities is shown in Table 9.

**Table 9:** The detailed information of the top 20 entities.

Entity^1^	Count^2^	Type^3^	Note
P08684	45	Enzyme	Referring to the gene CYP3A4
hsa05322	41	Pathway	
P02768	41	Carrier	
1576	41	Gene	Referring to the gene CYP3A4
hsa04740	40	Pathway	
1565	39	Gene	Referring to the gene CYP2D6
P11712	33	Enzyme	Referring to the gene CYP2C9
P10632	32	Enzyme	
1559	27	Gene	Referring to the gene CYP2C9
P08183	27	Transporter	Referring to the gene ABCB1
1544	26	Gene	Referring to the gene CYP1A2
P10635	26	Enzyme	Referring to the gene CYP2D6
1557	25	Gene	Referring to the gene CYP2C19
P05177	24	Enzyme	Referring to the gene CYP1A2
5243	21	Gene	Referring to the gene ABCB1
hsa04080	20	Pathway	
P33261	19	Enzyme	Referring to the gene CYP2C19
P20815	18	Enzyme	Referring to the gene CYP3A5
1577	17	Gene	Referring to the gene CYP3A5
hsa05332	17	Pathway	

^1^ Entity means the name of the entities in the knowledge graph.

^2^ Count means the number of drugs associated with that entity among the top 100 drugs.

^3^ Type means the category of the entity.

Among the top 20 entities, we observed seven entity pairs that correspond to the same genes: P08684 and gene 1576, P10635 and gene 1565, P11712 and gene 1559, P08183 and gene 5243, P05177 and gene 1544, P33261 and gene 1557, as well as P20815 and gene 1577. Taking the enzyme P08684 and gene 1576 as an example, P08684 represents the UniProt ID for the CYP3A4 protein, while 1576 is the NCBI Gene ID for the CYP3A4 gene. Both of these identifiers map to the same official gene name, namely CYP3A4. The CYP3A4 gene is translated into the CYP3A4 enzyme through transcription and translation processes. This discovery highlights that our MolFPKG-DILI model exhibits a high degree of consistency in integrating both gene-level and protein-level information. It is capable of effectively recognizing different molecular representations of the same biological entity, thereby demonstrating its robustness and comprehensiveness in handling complex biological data.

We conducted a literature review on selected entities and found substantial evidence supporting their associations with liver function or hepatotoxicity.

(1) P08684 and gene 1576 (CYP3A4): CYP3A4 is a cytochrome P450 monooxygenase predominantly expressed in the liver and small intestine, involved in the metabolism of sterols, steroid hormones, retinoids and fatty acids. Importantly, accumulating evidence demonstrates CYP3A4’s involvement in DILI through metabolic activation pathways. Kim et al. [40] reported that the CYP3A4-mediated metabolic process of the anticancer drug erlotinib gives rise to reactive intermediates, such as epoxides and quinone imines, which exert direct hepatotoxic effects. Their study further showed that CYP3A4 inducers exacerbate this toxicity by accelerating the production of these harmful metabolites. Similarly, Klarissa et al. [41] demonstrated that CYP3A4 inducers promote the metabolic activation of lapatinib, leading to increased generation of reactive toxic intermediates and subsequent hepatocellular damage.

(2) hsa05322: The hsa05322 pathway is associated with systemic lupus erythematosus (SLE), a multisystem autoimmune disorder. The management of SLE generally necessitates the use of immunosuppressive medications [42]. Significantly, drug-induced liver injury is a commonly encountered therapeutic complication in patients with SLE. Takahashi et al. [43] conducted a comprehensive analysis of 123 SLE patients presenting with hepatic dysfunction and identified pharmacological agents as the causative factor in approximately 30.9% of cases. Additionally, there is currently evidence that aspirin and thiopurine analogs can lead to liver function impairment in some SLE patients [44].

(3) P02768: P02768 refers to albumin, a principal plasma protein synthesized by the liver. It performs essential functions such as molecular transport, anti-oxidation, anti-inflammation, and the adjustment of capillary permeability, and is commonly used in the treatment of patients with liver cirrhosis and liver failure [45,46]. Albumin-related genes are highly expressed in the liver and are involved in the "heme biosynthesis" and "heme degradation" pathways, both of which are associated with the liver’s detoxification and metabolic functions. Research conducted by Sun et al. [46] indicates that various liver diseases are associated with impairments in albumin function, and dysfunction of albumin may be a new biomarker of early impairment in liver function.

These cases not only demonstrate that the model can effectively capture important biological relationships with pathophysiological foundations within the knowledge graph, but also show that the identified features have clear biological explanations. The MolFPKG-DILI model ensures predictive performance for hepatotoxicity classification tasks while also providing good decision transparency and interpretability.

## Conclusion

This study proposes a liver toxicity prediction model called MolFPKG-DILI, which utilizes three types of features: molecular fingerprints, molecular graphs, and knowledge graph, to classify drug-induced liver toxicity. The model incorporates an attention mechanism to fuse molecular fingerprints and molecular graph features, focusing on the most important molecular information. Subsequently, these fused features are input into a drug-centered knowledge graph, enabling the model to learn toxicity-related features from the complex graph network. The model performs well in identifying the presence or absence of liver toxicity, with an accuracy of 72.73%, and in distinguishing between strong and weak toxicity, with an accuracy of 75.81%. These results surpass a series of baseline algorithms.

Although our model achieves satisfactory performance, there are still some limitations to our work. Firstly, the RGCN module can only handle static graph information. This means that whenever we have new drugs or updated knowledge graph, we need to retrain and retest the model, which lacks scalability. In this study, the collection of experimental data is not extensive enough. We only obtained labels for the severity of liver toxicity as strong and weak, without further refinement of the severity levels or validation experiments on external datasets. Additionally, the model primarily uses molecular structural features and knowledge graph for predictions, lacking information such as dose-response data and pharmacokinetic parameters, which poses certain limitations in clinical applications.

The use of knowledge graph for predicting drug-induced liver toxicity provides a new direction in the field of liver toxicity prediction. In the future, we will continue to optimize the feature processing modules of this model to further improve its predictive performance in liver toxicity classification. Additionally, since the model is not limited to predicting liver toxicity alone, we will explore the use of knowledge graph to predict a range of drug properties. Our goal is to build a more versatile model that can accelerate the drug development process.

## Supporting information

S1 Fig Visualization of chemical properties of Most-DILI and Less-DILI drugs. (A) Tanimoto similarity heatmap of all compounds using Morgan fingerprint. (B) The t-SNE distribution of the compounds labeled Most-DILI and Less-DILI. (C) Physicochemical property distributions of compounds with Most-DILI and Less-DILI.
(PDF)

S1 Text The detailed information of baselines. (PDF)

S2 Text Analysis of comparative experiments on drug-drug similarity. (PDF)

S3 Text Model performance analysis of drugs with different ATC codes. (PDF)

S4 Text The procedure to generate the independent validation set. (PDF)

S1 Table The prediction results of drugs in the task of classifying DILI and No-DILI. (XLSX)

S2 Table The prediction results of drugs in the task of classifying Most-DILI and Less-DILI. (XLSX)
