# Supplementary figures and images for: Integrating multi-type features and knowledge graph for graded prediction of drug-induced liver injury in humans
Source: PLoS Comput Biol. 2026 Jul 14;22(7):e1013640. doi: 10.1371/journal.pcbi.1013640 (PMC13367694; doi:10.1371/journal.pcbi.1013640)

A

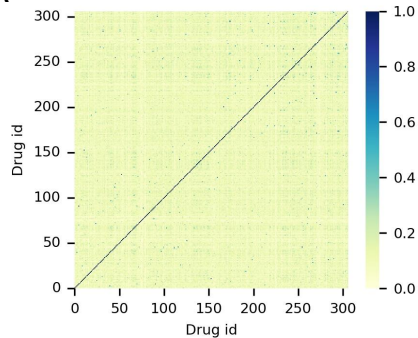

C

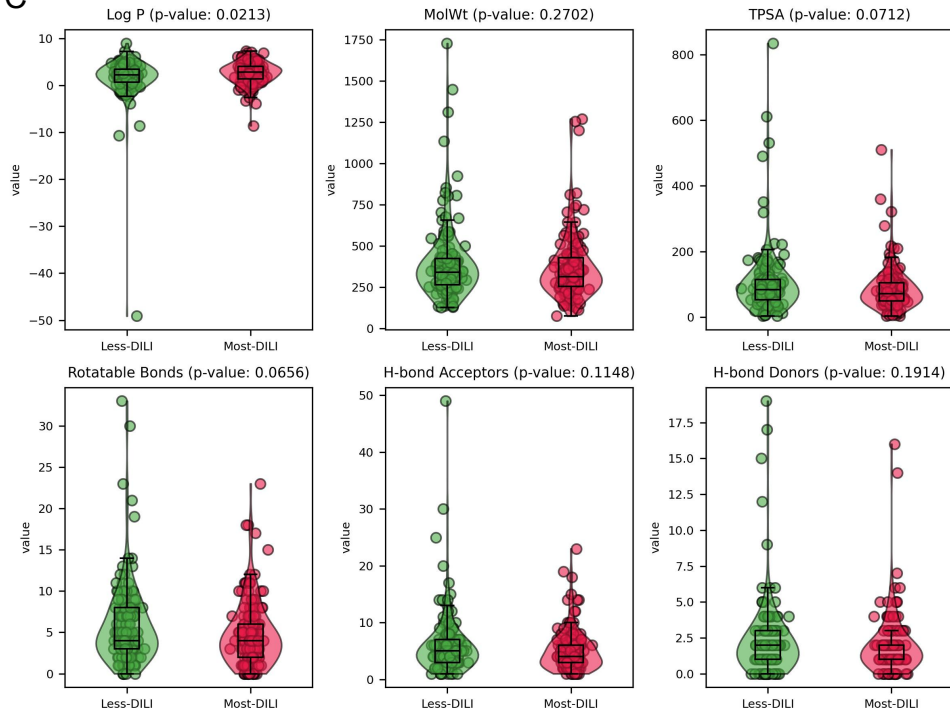

B

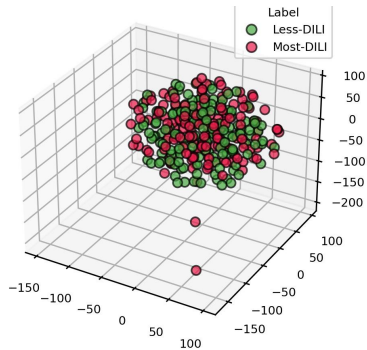

Supplement: S1 Fig — (A) Tanimoto similarity heatmap of all compounds using Morgan fingerprint. (B) The t-SNE distribution of the compounds labeled Most-DILI and Less-DILI. (C) Physicochemical property distributions of compounds with Most-DILI and Less-DILI. (PDF) [file pcbi.1013640.s001.pdf]
