# Supplementary material for: Integrating multi-type features and knowledge graph for graded prediction of drug-induced liver injury in humans
Source: PLoS Comput Biol. 2026 Jul 14;22(7):e1013640. doi: 10.1371/journal.pcbi.1013640 (PMC13367694; doi:10.1371/journal.pcbi.1013640)
Supplement: S1 Text — (PDF) [file pcbi.1013640.s002.pdf]

S1 Text. The detailed information of baselines.

The baseline models employed in this study can be categorized into four groups: (1) models utilizing only molecular fingerprint features, which include SVM (Support Vector Machine), RF (Random Forest), FPNN, DeepDILI [1], and 2020-DILI-CNN-MFE [2]; (2) models utilizing only molecular graph features, which include GCN (Graph Convolutional Network), GAT (Graph Attention Network) [3], GraphSAGE (Graph Sample and Aggregation) [4] and DILIGeNN [5]; (3) models combining both molecular fingerprint and molecular graph features, represented by FP-Graph; and (4) models using only knowledge graph, which include KGDNN [7], KG-SVM and KG-RF.

- SVM, RF: SVM is a supervised learning algorithm with the primary goal of finding the optimal hyperplane that maximizes the margin between different categories. SVM maps data points into a higher-dimensional space to enable the separation of classes. When dealing with non-linearly separable data, SVM utilizes kernel functions to transform the data into higher dimensions, allowing a linear hyperplane to be identified in this new space. RF is an ensemble learning algorithm that constructs multiple decision trees and aggregates their predictions through voting or averaging. The main advantage of RF is its ability to mitigate overfitting, as the voting results from multiple trees can smooth out the biases and variances present in individual decision trees. Additionally, RF supports feature importance evaluation, which provides insights into the contribution of each input feature towards the final predictions.

- FPNN: FPNN is essentially a multilayer perceptron (MLP) that learns from the given feature vectors through nonlinear transformations performed by multiple fully connected layers to obtain prediction results. In this study, we designed an FPNN model with four fully connected layers. The input data consists of concatenated features from three types of molecular fingerprints. The features are processed through activation functions at each layer, and the output from one layer serves as the input to the next layer, eventually producing the model's predictions. We employed backpropagation to optimize the model parameters by minimizing the error between the predicted results and the actual labels.

- DeepDILI [1]: The framework first inputs multi-source features related to DILI (Drug Induced Liver Injury) prediction (e.g., molecular structure, in vitro toxicity data) into the five base algorithms (KNN (k-Nearest Neighbor), LR (Logistic Regression), SVM, RF, XGBoost) respectively, completes independent training, and obtains the core outputs of each algorithm. Subsequently, these "intermediate prediction information" from different algorithms are used as input features of the neural network; the neural network's fully connected layers then learns adaptive weights for each base algorithm. Finally, through end-to-end learning of the neural network, the advantages and complementarity of the five algorithms are converted into unified "model-level representations."

- 2020-DILI-CNN-MFE [2]: The model is technically implemented based on molecular fingerprint embedding and convolutional neural network (CNN). First, it integrates multi-source compound data to divide a development set and an independent test set, and converts compounds into molecular fingerprints and generates binary vectors using cheminformatics tools. Then, drawing on the idea of word embedding in natural language processing (NLP), it transforms the binary vectors into vectors containing substructure indexes, pads zeros to unify their lengths, and converts them into continuous embedded matrices with structural and semantic information through a trainable lookup table. Finally, it constructs a CNN model including an embedding layer, a convolutional block, and a fully connected block to achieve DILI classification.

- GCN, GAT [3], GraphSAGE [4]: GCN models the structural information of graphs by aggregating the features of each node and its neighbors. Stacking two layers of graph convolution updates node feature representations, incorporating both individual and neighboring influences. GAT enhances this process by introducing an attention mechanism to selectively aggregate neighboring features, learning attention weights that allow for flexible handling of complex molecular graph data. In GAT, the first layer utilizes a multi-head attention mechanism to compute four independent attention weights, enriching the graph embeddings. The second layer performs compact feature extraction on the first layer's output to derive the final node embeddings. GraphSAGE improves computational efficiency by sampling a fixed number of neighboring nodes and aggregating their features, making it suitable for large-scale molecular graph data. The first layer captures local features and extends them to the current node's neighborhood, while the second layer aggregates these outputs over a larger scope, resulting in more globally-informed node representations. This layered approach allows GraphSAGE to effectively combine local and global information in molecular graphs.

- DILIGeNN [5]: The DILIGeNN model proposed in this study is based on GNN framework. Technically, it first extracts augmented graph features (such as interatomic bond lengths, Gasteiger partial charges,

total valence electrons, etc.) through a molecular optimization process, and then constructs a custom molecular graph dataset containing 3D spatial and electrostatic information. Next, four classic GNN architectures (GCN, GAT, GraphSAGE, Graph Isomorphism Network[6]) are used for model construction; a nested cross-validation framework is applied for hyperparameter optimization, combined with model reinitialization using multiple random seeds to reduce the impact of weight initialization differences, and a sequential warm-start strategy is used for model fine-tuning.

- FP-Graph: This method leverages both molecular fingerprint and molecular graph features. It employs FPNN and GCN to learn from the molecular fingerprint and molecular graph features, respectively, concatenates the processed features from the two modules, and feeds them into an MLP classifier consisting of four fully connected layers to obtain the final results.

- KGDNN [7]: This method relies on knowledge graph (KG). It uses the Node2vec algorithm to generate KG node sequences, and then applies the CBOW (Continuous Bag of Words) model to convert discrete nodes into vector embeddings that contain structural and semantic information. Finally, it builds a deep fully connected neural network, takes the concatenated embeddings of drugs and ADRs (Adverse Drug Reactions) as input, and trains the model with regularization mechanisms to realize binary classification prediction of drug-ADR associations. In this experiment, we referred to its processing method for knowledge graph. We used drug node vectors and input them into MLP for drug toxicity classification.

- KG-SVM, KG-RF: This method processes knowledge graph in the same way as KGDNN, and the classification methods are changed to SVM and RF.

References

[1] Li T, Tong W, Roberts R, Liu Z, Thakkar S. DeepDILI: deep learning-powered drug-induced liver injury prediction using model-level representation. Chemical research in toxicology. 2020;34(2):550–565.

[2] Nguyen-Vo TH, Nguyen L, Do N, Le PH, Nguyen TN, Nguyen BP, et al. Predicting drug-induced liver injury using convolutional neural network and molecular fingerprint-embedded features. ACS omega. 2020;5(39):25432–25439.

[3] Velickovic P, Cucurull G, Casanova A, Romero A, Lio P, Bengio Y, et al. Graph attention networks. stat. 2017;1050(20):10–48550.

[4] Hamilton W, Ying Z, Leskovec J. Inductive representation learning on large graphs. Advances in neural information processing systems. 2017;30.

[5] Lee T, Posma J. Improving Drug-Induced Liver Injury Prediction Using Graph Neural Networks with Augmented Graph Features from Molecular Optimisation. 2025;.

[6] Xu K, Hu W, Leskovec J, Jegelka S. How powerful are graph neural networks? arXiv preprint arXiv:181000826. 2018;.

[7] Joshi P, Masilamani V, Mukherjee A. A knowledge graph embedding based approach to predict the adverse drug reactions using a deep neural network. Journal of Biomedical Informatics. 2022;132:104122.
