# Supplementary material for: Integrating multi-type features and knowledge graph for graded prediction of drug-induced liver injury in humans
Source: PLoS Comput Biol. 2026 Jul 14;22(7):e1013640. doi: 10.1371/journal.pcbi.1013640 (PMC13367694; doi:10.1371/journal.pcbi.1013640)
Supplement: S2 Text — (PDF) [file pcbi.1013640.s003.pdf]

# S2 Text. Analysis of comparative experiments on drug-drug similarity.

In this study, we introduced drug-drug similarity to establish associations between drugs and enrich the network information in the knowledge graph (KG). This facilitated the updating of information between drug nodes and improved the accuracy of liver toxicity grading. The number of edges introduced into the KG varies depending on the defined similarity threshold, and the performance of the model also differs accordingly. Table 1 and 2 display the performance of the MolFPKG-DILI model under different similarity thresholds for various classification tasks. From the table, it can be observed that the performance of the model does not have a simple linear relationship with the similarity threshold. Instead, better performance is achieved when the similarity thresholds are set to 0.9, 0.8, and 0.5, and the performance in both toxicity and toxicity severity classification is not lower than that without the addition of drug-drug similarity edges. Overall, most indicators suggest that the model performs best when the similarity threshold is set to 0.8.

**Table 1. The model performance in classifying non-toxicity at different drug similarity thresholds.**

| Similarity threshold | Number of drug-drug edges | Accuracy | Precision | Sensitivity | Specificity | F1-Score |
|---|---|---|---|---|---|---|
| 1 | 0 | 0.7127 | 0.7255 | 0.7024 | 0.7235 | 0.7133 |
| 0.9 | 21 | 0.7236 | **0.7443** | 0.6976 | **0.7506** | 0.7195 |
| 0.8 | 40 | **0.7273** | 0.7408 | **0.7167** | 0.7383 | **0.7274** |
| 0.7 | 112 | 0.7176 | 0.7320 | 0.7048 | 0.7309 | 0.7173 |
| 0.6 | 231 | 0.7188 | 0.7349 | 0.7024 | 0.7358 | 0.7171 |
| 0.5 | 457 | 0.7200 | 0.7398 | 0.6976 | 0.7432 | 0.7177 |

**Table 2. The model performance in classifying strong and weak toxicity at different drug similarity thresholds.**

| Similarity threshold | Number of drug-drug edges | Accuracy | Precision | Sensitivity | Specificity | F1-Score |
|---|---|---|---|---|---|---|
| 1 | 0 | 0.7452 | 0.7219 | 0.8065 | 0.6839 | 0.7580 |
| 0.9 | 1 | 0.7452 | 0.7203 | 0.8065 | 0.6839 | 0.7579 |
| 0.8 | 2 | **0.7581** | **0.7369** | 0.8065 | **0.7097** | **0.7680** |
| 0.7 | 12 | 0.7452 | 0.7240 | 0.8000 | 0.6903 | 0.7568 |
| 0.6 | 26 | 0.7484 | 0.7272 | 0.8000 | 0.6968 | 0.7585 |
| 0.5 | 51 | 0.7516 | 0.7258 | **0.8129** | 0.6903 | 0.7643 |
