# Supplementary material for: Integrating multi-type features and knowledge graph for graded prediction of drug-induced liver injury in humans
Source: PLoS Comput Biol. 2026 Jul 14;22(7):e1013640. doi: 10.1371/journal.pcbi.1013640 (PMC13367694; doi:10.1371/journal.pcbi.1013640)
Supplement: S3 Text — (PDF) [file pcbi.1013640.s004.pdf]

S3 Text. Model performance analysis of drugs with different ATC codes.

The Anatomical Therapeutic Chemical (ATC) code is an official drug classification system proposed by the World Health Organization (WHO). The code consists of 7 characters, and the first letter categorizes drugs into 14 classes based on anatomical knowledge. Where, A represents Alimentary tract and metabolism, B represents Blood and blood forming organs, C represents Cardiovascular system, and D represents Dermatologicals, G represents Genito urinary system and sex hormones, H represents Systemic hormonal preparations, excluding sex hormones and insulins, J represents Antiinfective for systemic use, L represents Antineoplastic and immunomodulating agents, M represents Musculo-skeletal system, N represents Nervous system, P represents Antiparasitic products, insecticides and repellents, R represents Respiratory system, S represents Sensory organs and V represents Various.

In this study, we followed this classification rule to divide drugs into 14 categories and observed the toxicity grading results for each category. As shown in Fig 1a and 1b, each classification task includes 14 subgraphs corresponding to the 14 categories. The square area of each subgraph represents the magnitude of the corresponding indicator for that drug class. Detailed results can be found in Table 1.

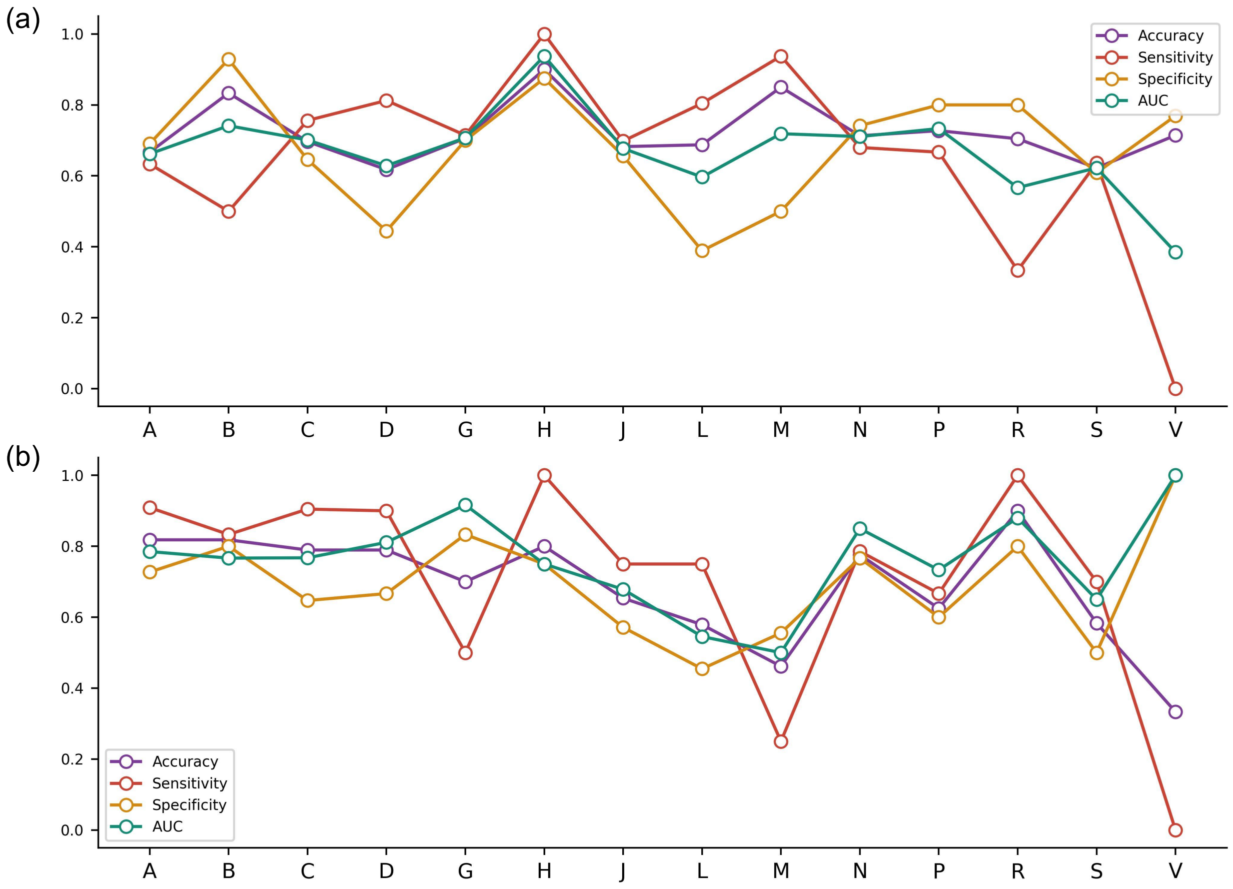

Figure 1 The model performance of drugs with different ATC codes. (a) is for DILI and No-DILI data, and (b) is for Most-DILI and Less-DILI data.

In terms of toxicity classification, category H shows significantly better predictive accuracy compared to other categories, with an accuracy of 0.9000, sensitivity of 1.0000, specificity of 0.8750, and AUC of 0.9375. The accuracy improvement for category H is nearly 19% compared to the results of the MolFPKG-DILI model on all drugs. Category H corresponds to hormonal drugs, indicating that the model performs well in classifying the hepatotoxicity of hormonal drugs. Regarding the strong and weak toxicity classification, Fig 1b shows that category R has the best results, and categories A and B also exhibit satisfactory performance. This suggests that the model effectively distinguishes between strong and weak toxicity for respiratory system, blood system, and gastrointestinal drugs. However, overall, the model's performance on category S and L drugs is average, indicating poorer recognition of hepatotoxicity for sensory system, anticancer, and immune drugs. Moreover, the model shows a sensitivity of 0 for category V drugs, which may be related to the fact that category V includes contrast agents, alexipharmacons, and other complex drugs. These drugs cannot be used as treatment

methods for specific organ diseases, and there is limited research on related hepatotoxicity, with a weak ability to identify hepatotoxicity.

Table 1. The model performance of drugs with different ATC codes.

(a) DILI vs No-DILI

Class	Accuracy	Precision	Sensitivity	Specificity	F1-Score	AUC
A	0.6667	0.5938	0.6333	0.6905	0.6129	0.6619
B	0.8333	0.6667	0.5000	**0.9286**	0.5714	0.7143
C	0.6966	0.6458	0.7561	0.6458	0.6966	0.7010
D	0.6176	0.5652	0.8125	0.4444	0.6667	0.6285
G	0.7059	0.6250	0.7143	0.7000	0.6667	0.7071
H	**0.9000**	0.6667	**1.0000**	0.8750	0.8000	**0.9375**
J	0.6824	0.7708	0.6981	0.6562	0.7327	0.6772
L	0.6875	0.7708	0.8043	0.3889	0.7872	0.5966
M	0.8500	**0.8824**	0.9375	0.5000	**0.9091**	0.7188
N	0.7130	0.6939	0.6800	0.7414	0.6869	0.7107
P	0.7273	0.8000	0.6667	0.8000	0.7273	0.7333
R	0.7045	0.3000	0.3333	0.8000	0.3158	0.5667
S	0.6222	0.6087	0.6364	0.6087	0.6222	0.6225
V	0.7143	0.0000	0.0000	0.7692	0.0000	0.3846

(b) Most-DILI vs Less-DILI

Class	Accuracy	Precision	Sensitivity	Specificity	F1-Score	AUC
A	0.8182	0.7692	0.9091	0.7273	0.8333	0.7851
B	0.8182	**0.8333**	0.8333	0.8000	0.8333	0.7667
C	0.7895	0.7600	0.9048	0.6471	0.8261	0.7675
D	0.7895	0.7500	0.9000	0.6667	0.8182	0.8111
G	0.7000	0.6667	0.5000	0.8333	0.5714	0.9167
H	0.8000	0.5000	**1.0000**	0.7500	0.6667	0.7500
J	0.6538	0.6000	0.7500	0.5714	0.6667	0.6786
L	0.5789	0.5000	0.7500	0.4545	0.6000	0.5455
M	0.4615	0.2000	0.2500	0.5556	0.2222	0.5000
N	0.7759	0.7586	0.7857	0.7667	0.7719	0.8500
P	0.6250	0.5000	0.6667	0.6000	0.5714	0.7333
R	**0.9000**	**0.8333**	**1.0000**	0.8000	**0.9091**	0.8800
S	0.5833	0.5000	0.7000	0.5000	0.5833	0.6500
V	0.3333	0.0000	0.0000	**1.0000**	0.0000	**1.0000**
