# Supplementary material for: Integrating multi-type features and knowledge graph for graded prediction of drug-induced liver injury in humans
Source: PLoS Comput Biol. 2026 Jul 14;22(7):e1013640. doi: 10.1371/journal.pcbi.1013640 (PMC13367694; doi:10.1371/journal.pcbi.1013640)
Supplement: S4 Text — (PDF) [file pcbi.1013640.s005.pdf]

S4 Text. The procedure to generate the independent validation set.

The independent validation set of drugs was derived from the remaining samples in the DILIrank and DILIst, with the screening process as follows:

Initially, we focused on the DILIrank database, filtering through 254 drugs labeled as "Ambiguous DILI-concern". After excluding samples that were also present in the DILIst database, 129 drugs remained. We further eliminated drugs without corresponding SMILES structures, resulting in 116 candidate drugs. To clarify their hepatotoxicity characteristics, we retrieved evidence of hepatotoxicity from the LiverTox database, which yielded 35 drugs with documented evidence. According to the grading criteria of LiverTox, drugs with a Likelihood score of D or E were excluded due to uncertain hepatotoxicity evidence (33 drugs), leaving a final count of 2 drugs (glatiramer acetate and flurbiprofen). Glatiramer acetate was excluded due to the inability to obtain its association with other entities, resulting in the retention of only flurbiprofen, which was classified as Less-DILI. Subsequently, we processed 307 DILI drugs from the DILIst database that were not included in DILIrank. Upon searching the LiverTox database, we found 32 documented drugs. After excluding samples with a Likelihood score of D or E, 12 drugs remained. Combining the hepatotoxicity evidence from LiverTox with the classification standards from the DILIrank dataset, we further delineated toxicity levels and ultimately identified 6 Less-DILI drugs and 6 Most-DILI drugs.

Additionally, to supplement our dataset with No-DILI drugs, we integrated updated data from drug-related databases with the No-DILI drugs from the DILIst database. Given that the database versions used in previous experiments (as of September 2023) had updates, this study adopted knowledge graph from DGIdb (2024 Dec version) and DrugBank (2025-01-02 version). After rematching with the No-DILI drugs in DILIst, we identified an additional 4 usable No-DILI drugs.

In summary, the independent validation set comprises 4 No-DILI drugs and 13 DILI drugs (7 of which are classified as Less-DILI and 6 as Most-DILI). Detailed information can be found in Table 1.

Table 1. The details of independent validation set.

Label	Count	Drug List
No-DILI	4	colistimethate, penicillin v, polymyxin b sulfate, gadobenate dimeglumine
Less-DILI	7	buprenorphine, desflurane, flurbiprofen , plicamycin, rivaroxaban, tibolone, trabectedin
Most-DILI	6	abiraterone, boceprevir, crizotinib, floxuridine, halothane, telaprevir
